# Editors’ choice: The four most valued articles published in the *European Journal of General Practice* in 2019

**DOI:** 10.1080/13814788.2020.1761181

**Published:** 2020-05-13

**Authors:** Jelle Stoffers, On behalf of the Editorial Board of the European Journal of General Practice

**Affiliations:** Department of Family MedicineCare and Public Health Research Institute (CAPHRI) Maastricht University Maastricht, The Netherlands

In 2019, the EJGP published 21 original articles, 3 systematic reviews and 4 background and opinion papers, including the first part of a Series on eHealth [1]. Authors came from the Netherlands, Spain, Belgium, Denmark, Finland, France, Ireland, Israel, Poland, the Republic of Srpska, Bosnia and Herzegovina, Sweden and Switzerland. In contrast, three papers had an international group of authors. Besides, we published four Editorials, one invited commentary and one Letter to the Editor, as well as a selection of abstracts from the EGPRN conference in Tampere of May 2019 [2].

Like previous years, the EJGP editors evaluated the EJGP’s last year’s volume. They each picked three articles they found most valuable and of which they established the scientific quality as ‘good’. Overall, the highest-ranking paper was the well-performed systematic review on clinical leadership by GPs in interprofessional primary care teams by Minke Nieuwboer and colleagues [3].

They analysed the relationship between clinical leadership and the implementation of integrated primary care, and what leadership skills are essential for integrated primary care practice. Physicians seemed to be the most adequate leaders. Leaders’ relational and organisational skills, as well as process-management and change-management skills, improved care integration. The authors concluded that we need a more profound knowledge of leadership skills to implement integrated-care effectively and to support leaders in developing the necessary skills.

Other high-ranking articles were an original paper on transitional safety incidents [4], an original paper on diagnostic errors in primary healthcare and emergency departments [5], and a systematic review of factors associated with the identification of child mental health problems in primary care [6].

The EJGP editors congratulate our colleagues with their valuable contributions to the scientific literature underpinning our daily practice. Furthermore, we thank all authors who published in the EJGP in 2019 for sharing their work with our readers.
